# Electrochemical and Fluorescence MnO_2_-Polymer Dot Electrode Sensor for Osteoarthritis-Based Peroxisomal β-Oxidation Knockout Model

**DOI:** 10.3390/bios14070357

**Published:** 2024-07-22

**Authors:** Akhmad Irhas Robby, Songling Jiang, Eun-Jung Jin, Sung Young Park

**Affiliations:** 1 Chemical Industry Institute, Korea National University of Transportation, Chungju 27469, Republic of Korea; irhasakhmad@ut.ac.kr; 2 Department of Chemical & Biological Engineering, Korea National University of Transportation, Chungju 27469, Republic of Korea; 3 Integrated Omics Institute, Wonkwang University, Iksan 54538, Republic of Korea; jsl91800@wku.ac.kr; 4 Department of Biological Sciences, College of Health Sciences, Wonkwang University, Iksan 54538, Republic of Korea

**Keywords:** osteoarthritis, coenzyme A, chondrocyte cell, polymer dot, electrochemical sensor, fluorescence sensor

## Abstract

A coenzyme A (CoA-SH)-responsive dual electrochemical and fluorescence-based sensor was designed utilizing an MnO_2_-immobilized-polymer-dot (MnO_2_@D-PD)-coated electrode for the sensitive detection of osteoarthritis (OA) in a peroxisomal β-oxidation knockout model. The CoA-SH-responsive MnO_2_@D-PD-coated electrode interacted sensitively with CoA-SH in OA chondrocytes, triggering electroconductivity and fluorescence changes due to cleavage of the MnO_2_ nanosheet on the electrode. The MnO_2_@D-PD-coated electrode can detect CoA-SH in immature articular chondrocyte primary cells, as indicated by the significant increase in resistance in the control medium (R_24h_ = 2.17 MΩ). This sensor also sensitively monitored the increase in resistance in chondrocyte cells in the presence of acetyl-CoA inducers, such as phytol (Phy) and sodium acetate (SA), in the medium (R_24h_ = 2.67, 3.08 MΩ, respectively), compared to that in the control medium, demonstrating the detection efficiency of the sensor towards the increase in the CoA-SH concentration. Furthermore, fluorescence recovery was observed owing to MnO_2_ cleavage, particularly in the Phy- and SA-supplemented media. The transcription levels of OA-related anabolic (*Acan*) and catabolic factors (*Adamts5*) in chondrocytes also confirmed the interaction between CoA-SH and the MnO_2_@D-PD-coated electrode. Additionally, electrode integration with a wireless sensing system provides inline monitoring via a smartphone, which can potentially be used for rapid and sensitive OA diagnosis.

## 1. Introduction

Osteoarthritis (OA) is a major degenerative disease that causes joint pain and movement disability and affects public health worldwide [1,2,3]. OA is a systemic and slow-progressing chronic disease responsible for joint destruction, including articular cartilage, knee meniscus, and ligament degradation, followed by painful joint deformities [4,5,6]. Patients with OA have higher levels of free fatty acids (FFAs), which are synthesized from acetyl-CoA via glycolysis and the tricarboxylic acid (TCA) cycle [7,8,9]. Acetyl-CoA is also a byproduct of fatty acid β-oxidation, and impaired peroxisomal β-oxidation contributes to acetyl-CoA accumulation, leading to cartilage degradation [10,11,12]. Peroxisomal β-oxidation breaks down fatty acids with very long chains into smaller molecules that are imported into mitochondrial β-oxidation [13,14,15]. It has been suggested that impaired peroxisomal β-oxidation contributes to the accumulation of acetyl-CoA in cartilage cells and leads to cartilage degeneration [16,17,18,19]. Moreover, in OA, increased glycolysis produces more acetyl-CoA catalyzed by coenzyme A (CoA-SH), and this accumulated acetyl-CoA can be used as an indicator of OA [20,21,22]. Owing to the crucial role of CoA-SH in OA pathogenesis, the high levels of CoA-SH expressed in OA chondrocytes can be used as a marker for OA progression [23,24,25]. Therefore, designing a coenzyme A/CoA-SH-responsive sensor would be beneficial for the easy monitoring of OA progression.

In the field of biosensing, polymer dots (PDs) derived from functionalized polymers have been extensively explored because of their outstanding properties, such as water solubility, biocompatibility, fluorescence, electroconductivity, and facile functionalization with various moieties and compounds [26,27,28,29]. Owing to these benefits, PDs can be utilized to design dual electrochemical and fluorescence-based biosensors [30,31]. Importantly, the versatility of PDs functionalized with a wide range of moieties and compounds would produce specific characteristics, such as stimuli-responsive properties, which can be used to target specific analytes [32,33,34,35,36]. For example, by modifying PDs with manganese oxide (MnO_2_) nanosheets, redox-responsive hybrid nanoparticles can be obtained and utilized for detecting redox-responsive species by measuring changes in conductivity and fluorescence before and after detection [30,31]. Considering that redox-active CoA-SH is a prominent species in OA, a CoA-SH-responsive sensor with a dual indicator of electroconductivity and fluorescence changes can be fabricated using the PD-MnO_2_-based sensor to monitor OA progression. This sensor is expected to produce distinct electroconductivity and fluorescence depending on the concentration of CoA-SH in OA chondrocytes, including the enhanced CoA-SH concentration in the presence of acetyl-CoA inducers, such as phytol (Phy) and sodium acetate (SA) [37,38,39].

Herein, we developed a dual electrochemical and fluorescence-based sensor utilizing a coenzyme A-responsive MnO_2_@D-PD-coated electrode for the simple and sensitive detection of OA chondrocytes. OA chondrocyte cell detection by this sensor relies on the redox reaction between CoA-SH and MnO_2_@D-PD on the electrode surface, which triggers the cleavage of the MnO_2_ nanosheet. The decomposition of MnO_2_ further changes the conductivity of the sensor and affects the fluorescence recovery of D-PD, indicating the sensitive detection of CoA-SH. The as-synthesized electrode was used to detect CoA-SH in immature articular chondrocyte (iMAC) primary cells and the enhanced CoA-SH levels in the presence of Phy- and SA-supplemented media. Moreover, a smartphone-based wireless sensing system was arranged with an MnO_2_@D-PD-coated electrode to provide the simple and user-friendly monitoring of CoA-SH for practical applications. By designing this sensor, we expected that the MnO_2_@D-PD-coated electrode would offer a sensitive and simple monitoring approach for OA progression based on the accumulation of CoA-SH, as the detection of elevated CoA-SH concentration has not been explored previously. Also, this OA sensor can be potentially used to support future clinical diagnostics and therapy for OA.

## 2. Materials and Methods

### 2.1. Materials

Sodium alginate, dopamine hydrochloride, coenzyme A (CoA-SH), poly (allylamine hydrochloride) (PAH), *N*-hydroxysuccinimide (NHS), ethylcarbodiimide hydrochloride (EDC), trizma base, potassium permanganate (KMnO_4_), and 2-(*N*-morpholino) ethanesulfonic acid were purchased from Sigma-Aldrich (St. Louis, MO, USA). Silicon (Si) wafer (P-type) was purchased from Silicon Technology Corporation, Republic of Korea. Phosphate buffered saline (PBS) was purchased from Bioneer Corp, (Daejeon, Republic of Korea). Fetal bovine serum (FBS), trypsin-ethylenediaminetetraacetic acid (trypsin-EDTA, 0.03% *w*/*v*), penicillin–streptomycin, Dulbecco’s modified Eagle’s medium (DMEM), and Roswell Park Memorial Institute (RPMI)−1640 medium were obtained from Gibco BRL (New York, NY, USA). Annexin-V-FITC and propidium iodide (PI) cell staining dyes were procured from Life Technologies (Carlsbad, CA, USA).

### 2.2. Characterizations

Absorbance spectra were analyzed using a UV-Vis spectrophotometer (Optizen 2120UV, Mecasys, Republic of Korea). The nanoparticle size was measured by using a dynamic light scattering (DLS) spectrometer (Zetasizer Nano, Malvern Panalytical, Herrenberg, Germany). Photoluminescence (PL) properties were evaluated using an L550B luminescence spectrometer (Perkin Elmer, Waltham, MA, USA), and the confocal images were captured using an ECLIPSE Ti2-E confocal microscope (Nikon, Tokyo, Japan). Surface images were captured using a scanning electron microscope (SEM, JSM-6700F, JEOL, Tokyo, Japan). The electrochemical properties were confirmed using an electrochemical impedance spectrometer (EIS; CS350, CorrTest Instrument, Wuhan, China) and sourcemeter (Keithley 2450, Tektronik, Beaverton, OR, USA). The wireless system consisted of an Arduino Uno micro-controller (ATmega328P Processor, Microchip, Chandler, AZ, USA) as the sensing part, a smartphone as the real-time data display, and a Bluetooth module (AppGosu) as the wireless communication bridge between Arduino Uno and the smartphone.

### 2.3. Synthesis of Dopamine-Conjugated Polymer Dots (D-PDs)

The D-PD nanoparticle was synthesized based on previous work [40]. Briefly, alginate-dopamine was firstly synthesized by adding EDC (0.38 g) and NHS (0.23 g) into the 100 mL alginate solution (5 g of alginate in PBS 2X pH 4) and allowed to react for 2 h at room temperature. Subsequently, dopamine (0.19 g) was added into the solution and reacted for another 24 h at room temperature. The solution was dialyzed (MWCO: 3.5 kDa) and freeze-dried. For D-PD synthesis, the obtained alginate-dopamine (1 g) was dissolved in 50 mL of DDW in the Teflon-lined hydrothermal reactor and subjected to a reaction in the oven at 180 °C for 8 h. Afterwards, the solution was freeze-dried to obtain D-PD.

### 2.4. Synthesis of D-PD-Conjugated Manganese Oxide Nanosheet (MnO_2_@D-PD)

The PAH-MnO_2_ nanosheet was synthesized by mixing 30 mg of PAH (in 6 mL DDW) into a 30 mL KMnO_4_ solution (contain 23.7 mg of KMnO_4_). Subsequently, 60 mL of MES solution (0.1 M, pH 6.0) was added into that solution and sonicated for 2 h. The solution was then dialyzed (MWCO: 3.5 kDa) and freeze dried. The as-synthesized PAH-MnO_2_ was then conjugated with D-PD via the EDC/NHS reaction. The D-PD solution (1 g in 100 mL of PBS 2X pH 3.8) was reacted with EDC (24.1 mg) and NHS (11.2 mg) for 2 h at room temperature. Subsequently, PAH-MnO_2_ (100 mg) was mixed into the solution and stirred for 24 h at room temperature. The solution was then dialyzed (MWCO: 3.5 kDa) and freeze-dried to obtain MnO_2_@D-PD.

### 2.5. Fabrication of MnO_2_@D-PD-Coated Electrode and Coated PET Surface

The electrochemical sensor was designed by coating MnO_2_@D-PD onto the electrode surface via a dip-coating process [33]. In brief, MnO_2_@D-PD (2 mg/mL) was dissolved in TBS pH 8.5, followed by soaking the Si wafer (1 × 1 cm) into that solution for 12 h. The MnO_2_@D-PD-coated Si wafer was then washed using DDW and dried prior to use for electrochemical-based detection. For the fluorescence study, MnO_2_@D-PD was coated onto the PET surface (1 × 1 cm) by using the same procedure as mentioned above.

### 2.6. Electrochemical Sensing of CoA-SH by MnO_2_@D-PD-Coated Electrode

The performance of the MnO_2_@D-PD-coated electrode for CoA-SH detection was examined using a sourcemeter (2-electrode set-up) and EIS (3-electrode set-up). The MnO_2_@D-PD-coated electrode was incubated with a CoA-SH solution in various concentrations (1,5, 10 mM) for 12 h. The electrode was then washed with DDW and dried prior to use. For the sourcemeter measurement, the CoA-SH-treated MnO_2_@D-PD-coated electrode was connected to the 2-electrode DC resistance system, and the resistance was measured with the sourcemeter. For the EIS measurement, the CoA-SH-treated MnO_2_@D-PD-coated electrode was set as a working electrode and incubated with cell solutions, with Ag/AgCl, a Pt wire, and PBS pH 7.4 used as a reference electrode, counter electrode, and electrolyte, respectively. The impedances were measured in the frequency range of 10^4^ – 10^−1^ Hz with a DC potential of −1.2 V vs the reference electrode and obtained as a Nyquist plot (*Z*′ vs. −*Z*″). All measurements were conducted at room temperature (25 °C).

### 2.7. Wireless CoA-SH Sensing of MnO_2_@D-PD-Coated Electrode

A wireless sensing system was constructed utilizing the 2-electrode DC resistance measurement system consisting of a microcontroller unit (Arduino Uno), a Bluetooth module (AppGosu), and a smartphone. The MnO_2_@D-PD-coated electrode was connected to a micro-controller and a Bluetooth module using alligator clips. The obtained resistance values were transmitted and displayed on a smartphone as graphical data (resistance graph) by activating the Bluetooth connection.

### 2.8. Fluorescence-Based Detection of CoA-SH

The MnO_2_@D-PD-coated PET surface was incubated with various CoA-SH solutions (1,5, 10 mM) for 12 h. Subsequently, the coated PET surface was washed with DDW and dried. The MnO_2_@D-PD-coated PET surface was then put under a confocal microscope for observing fluorescence recovery.

### 2.9. In Vitro Detection Performance of MnO_2_@D-PD-Coated Electrode Towards OA Articular Chondrocyte Cells

Primary cultured immature articular chondrocytes (iMACs) from mouse articular cartilage were isolated from postnatal day 5 to 6 mice via dissection of the tibial plateaus and femoral condyles. Cartilage was digested with a 3 mg/mL collagenase D (Roche, 11088858001, Basel, Switzerland) solution for 45 min and transferred to a new culture dish containing a 0.5 mg/mL collagenase D solution and incubated overnight at 37 °C. After filtering the digested cartilage through a 70 μm cell strainer, primary chondrocytes were cultured with low glucose (1 g/L) DMEM media supplemented with 10% FBS and penicillin–streptomycin at 37 °C in the presence of 5% CO_2_ for 5 days. The cultured cells were than incubated with an MnO_2_@D-PD-coated electrode in control media (Control), phytol-supplemented media (Phy), and sodium acetate-supplemented media (SA) for 12 h and 24 h at 37 °C prior to electrochemical and fluorescence-based analysis.

### 2.10. Quantitative Real-Time (qRT)-PCR

iMACs were treated either with Phy or SA, and total RNA was isolated, and the concentration and purity of the isolated RNA were assessed using a spectrophotometer. Complementary DNA (cDNA) was synthesized from 1 µg of total RNA using a reverse transcription kit following the manufacturer’s instructions. Real-time PCR was performed using a SYBR Green PCR master mix on a real-time PCR system. The relative expression levels of peroxisome-related genes, reported previously [9], were determined. Each sample was run in triplicate, and β-actin was used as the internal control for normalization. The cycling conditions were as follows: initial denaturation at 95 °C for 10 min, followed by 40 cycles of denaturation at 95 °C for 15 s, annealing at the optimal temperature for each primer set for 30 s, and extension at 72 °C for 30 s. A melt curve analysis was performed at the end of each PCR to verify the specificity of the amplification.

### 2.11. Bioinformatics Analysis

Public RNA-Seq data from the Gene Expression Omnibus (GEO) database (GSE162510 for IL1β-treated human chondrocytes, GSE162510 for IL1β-treated human chondrocytes, and GSE75181 for osteoarthritis patient chondrocytes) were analyzed to identify the signaling pathway affected during the pathogenesis of osteoarthritis (OA). To gain insights into the biological functions affected by the downregulated genes in osteoarthritis conditions, pathway enrichment analysis was conducted using the DAVID (Database for Annotation, Visualization, and Integrated Discovery) bioinformatics tool.

## 3. Results and Discussion

### 3.1. Design of MnO_2_@D-PD-Coated Electrode for CoA-SH Detection

**Figure 1:**
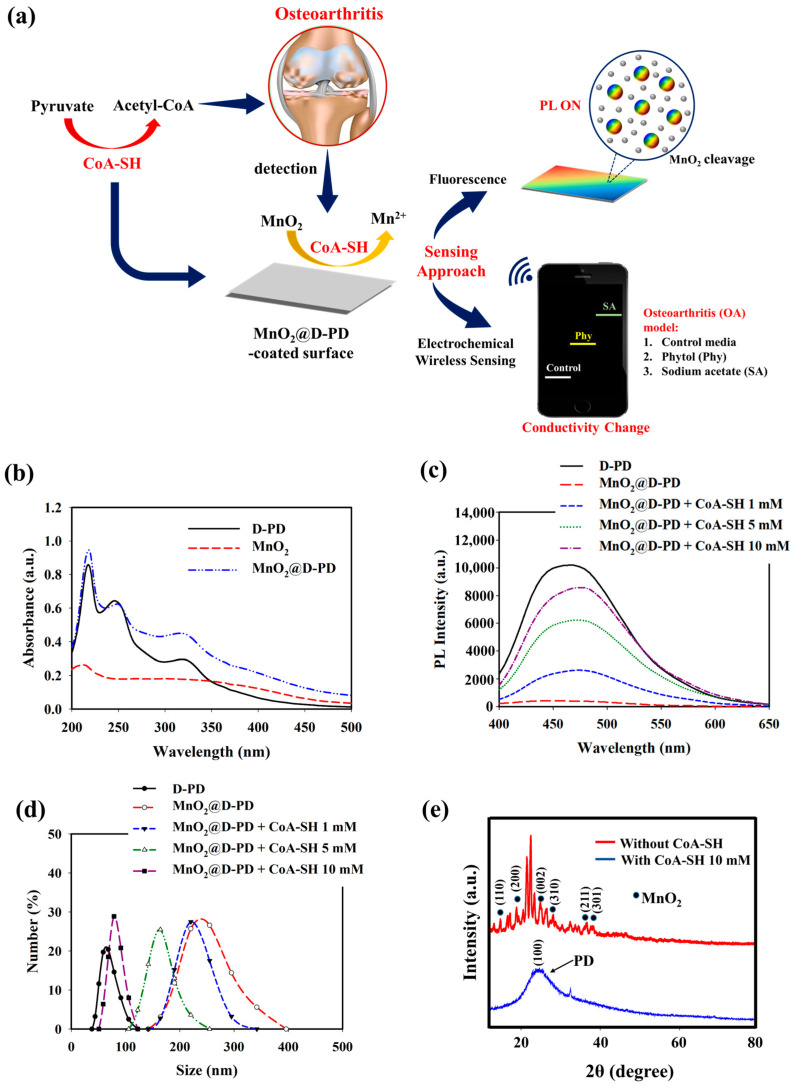
(**a**) Illustration of MnO_2_@D-PD-coated electrode fabrication and application for CoA-SH sensing. (**b**) UV-vis spectra, (**c**) PL spectra, (**d**) DLS profiles, and (**e**) XRD analysis of MnO_2_@D-PD nanoparticles in the absence and presence of CoA-SH.

A simple and sensitive approach for detecting OA was designed by fabricating a dual electrochemical and fluorescence-based MnO_2_@D-PD-coated electrode that utilizes the interaction between redox-responsive MnO_2_@D-PD and CoA-SH expressed in OA based on peroxisomal β-oxidation. Initially, MnO_2_@D-PD was synthesized by conjugating D-PD with a PAH-MnO_2_ nanosheet via EDC/NHS coupling, which was confirmed by quenching the fluorescence of D-PD owing to the Forster resonance energy transfer (FRET) phenomenon after successful conjugation with PAH-MnO_2_ [31]. The as-synthesized MnO_2_@D-PD was then coated onto a substrate, such as an Si wafer, utilizing the catechol moieties in D-PD. Quantifying the redox interaction between CoA-SH and the MnO_2_@D-PD-coated electrode, which results in the cleavage of the MnO_2_ nanosheets on the electrode, can be utilized to detect OA. This MnO_2_ breakage affects the fluorescence recovery of D-PD owing to the reduced FRET effect and changes in the conductivity of the sensor; this indicates the sensitivity of the MnO_2_@D-PD-coated electrode towards CoA-SH levels [31]. By utilizing these changes, the designed electrode is expected to sensitively detect CoA-SH in immature murine articular chondrocyte (iMAC) cells. For further practical applications, a smartphone-based wireless sensing module can be combined with an MnO_2_@D-PD-coated electrode to display the output of electronic signals on a smartphone upon CoA-SH detection (Figure 1a).

The optical properties of the synthesized MnO_2_@D-PD nanosheets were investigated using UV-Vis and PL spectroscopy. The absorbance peaks at 250 and 330 nm found in MnO_2_@D-PD corresponded to π–π* transition of the aromatic catechol ring in D-PD, while the peak at 400 nm was assigned to absorption by the MnO_2_ nanosheet, confirming the successful conjugation of MnO_2_ with D-PD (Figure 1b) [31,41,42]. The PL spectra further confirmed the conjugation of the MnO_2_ nanosheets with D-PD, as indicated by the FRET-induced fluorescence quenching in MnO_2_@D-PD compared with the fluorescence intensity of D-PD (Figure 1c) [31]. Subsequently, fluorescence recovery was observed when MnO_2_@D-PD was reacted with different concentrations of CoA-SH; the fluorescence intensities increased with higher concentrations of CoA-SH, indicating the decomposition efficacy of MnO_2_ nanosheets into Mn^2+^, leading to a reduced FRET effect. The cleavage of MnO_2_ in MnO_2_@D-PD was confirmed via DLS measurements before and after CoA-SH treatment. The particle sizes of MnO_2_@D-PD were found to be 253.40 nm and were gradually decreased to 223.75, 164.17, and 85.62 nm after treatment with CoA-SH at concentrations of 1, 5, and 10 mM, respectively, owing to the cleavage of the MnO_2_ nanosheet (Figure 1d). In addition, XRD analysis revealed the disappearance of MnO_2_ diffraction with a clear appearance of broad D-PD diffraction around 25° after CoA-SH treatment, confirming the redox responsiveness of the synthesized MnO_2_@D-PD towards CoA-SH (Figure 1e).

Owing to the availability of adhesive catechol moieties, the MnO_2_@D-PD nanosheets can be easily coated on the surfaces of various substrates to fabricate MnO_2_@D-PD-coated sensors. The coatability of MnO_2_@D-PD was evaluated based on water contact angle measurements of the Si wafer and PET coated with MnO_2_@D-PD (Figure S1a,b). Compared with the bare Si wafer (29.8°) and bare PET surface (67.3°), the contact angles of the Si wafer and PET coated with MnO_2_@D-PD changed to 42.3° and 46.9°, respectively. When CoA-SH was introduced onto both coated surfaces, gradual changes in the contact angle were observed (47.2°, 51.4°, and 54.1° for coated Si wafer; 49.3°, 53.4°, and 57.4° for coated PET) after treatment with 1, 5, and 10 mM CoA-SH, respectively. The increase in the contact angle affected by CoA-SH up to points close to the D-PD-coated surface contact angle (coated Si wafer = 58.8°; coated PET = 60.9°) demonstrates the decomposition of MnO_2_ nanosheets on the coated surface. In addition to compatibility, it is necessary to evaluate the coating stability under certain conditions, such as in cell media and ethanol solutions, because the electrode surface will be used for cell detection and subjected to sterilization. As shown in Figure S2a, the contact angle of the MnO_2_@D-PD-coated Si wafer remained unchanged, even after contact with the RPMI medium and ethanol solution for 12 h (± 42.0°). This coating stability can also be observed from the insignificant changes in the resistance before and after exposure to RPMI and ethanol solutions (Figure S2b).

**Figure 2:**
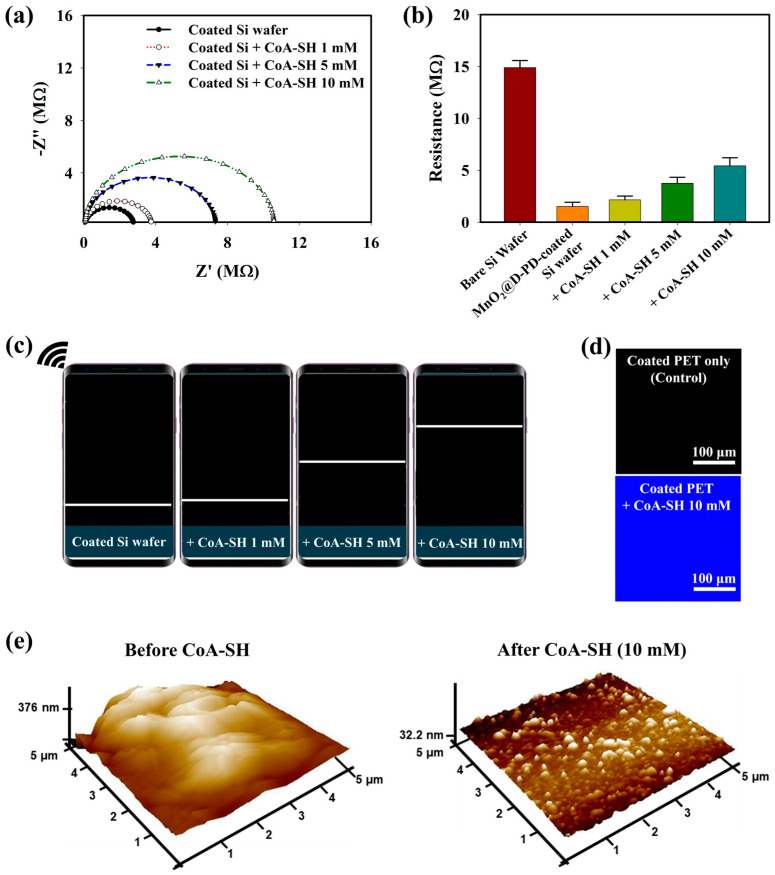
(**a**) EIS spectra, (**b**) sourcemeter measurement, and (**c**) wireless sensing profiles (shown as resistance graph) of MnO_2_@D-PD-coated Si wafer in the presence of various concentrations of CoA-SH. The effect of CoA-SH (10 mM) on the (**d**) fluorescence of the coated PET surface, and (**e**) surface morphology/AFM profile of MnO_2_@D-PD-coated Si wafer.

The interaction between CoA-SH and the MnO_2_@D-PD-coated electrode was observed electrochemically based on the change in the sensor resistance with various concentrations of CoA-SH. As demonstrated by the EIS spectra in Figure 2a, the resistance of the MnO_2_@D-PD-coated electrode gradually rose with an increasing CoA-SH concentration (1, 5, and 10 mM). This correlation was further evaluated using sourcemeter measurements, which showed that the cleavage of MnO_2_ by CoA-SH altered the resistance between before (1.50 MΩ) and after treatment with 1, 5, and 10 mM of CoA-SH (2.15, 3.75, and 5.42 MΩ, respectively) (Figure 2b). Electrochemical studies utilizing wireless sensing systems also confirmed a conductivity change in the MnO_2_@D-PD-coated electrode by showing rising patterns of electronic signals (displayed on a smartphone as a resistance graph) with increasing CoA-SH concentrations, revealing the sensitivity of the MnO_2_@D-PD-coated electrode towards CoA-SH (Figure 2c). The fluorescence of the MnO_2_@D-PD-coated electrode was also affected by the presence of CoA-SH. The confocal images in Figure 2d show fluorescence recovery after detecting CoA-SH from quenched emission to bright blue emission. This result indicates a decrease in the FRET effect as MnO_2_ is cleaved, which recovers the fluorescence of D-PD on the surface. The change in the surface morphology caused by CoA-SH was further observed via atomic force microscopy (AFM) analysis. As shown in Figure 2e, AFM profiles clearly showed distinct surface roughness of the MnO_2_@D-PD-coated electrode before and after the detection of CoA-SH, with surface thicknesses being approximately 376.0 nm and 32.2 nm, respectively. Furthermore, to assess the possible interferences from species contained in the media during CoA-SH detection, the selectivity test was conducted using various interfering species (glucose, lactose, bovine serum, PO_4_^3−^, and Na^+^). The results showed that the resistance of the MnO_2_@D-PD-coated electrode was unchanged even after incubation with each interference, while the significant increase in resistance occurred only after treatment with CoA-SH, confirming the selectivity of the as-synthesized sensor (Figure S3). 

### 3.2. In Vitro Detection Performance of MnO_2_@D-PD-Coated Electrode Towards OA Chondrocyte Cells in the Presence of Supplemented Phy and SA

**Figure 3:**
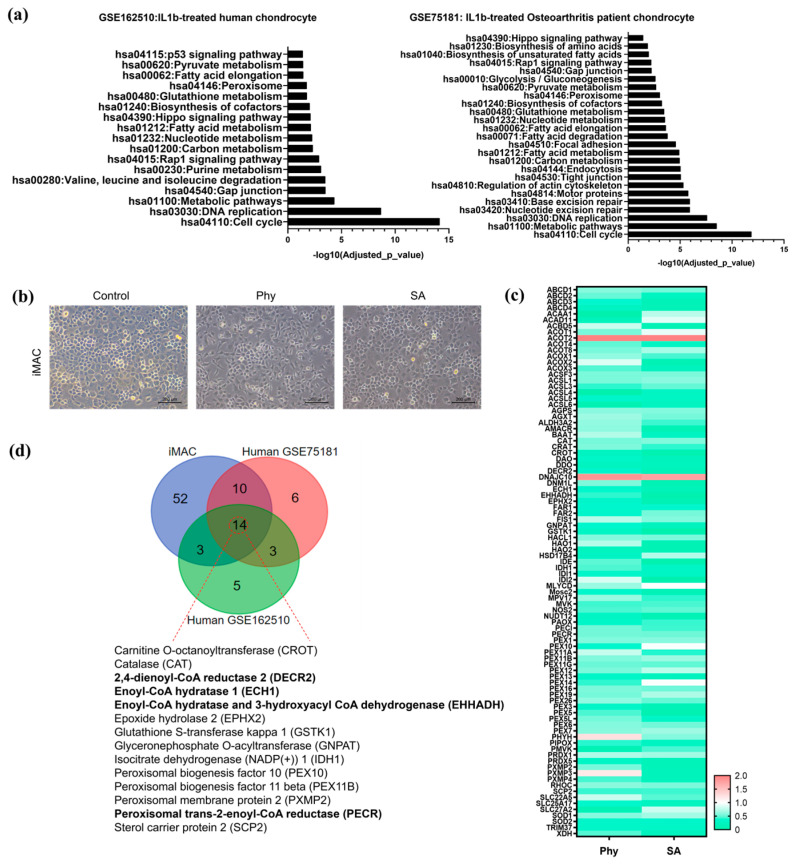
(**a**) Pathway enrichment analysis of downregulated genes in IL1β-treated human chondrocytes (GSE162510) and osteoarthritis patient chondrocytes (GSE75181) with key affected biological processes and pathways and corresponding *p*-values. (**b**) Bright field image of iMACs treated with Phy and SA, compared to controls. (**c**) The fold-change of peroxisome-related genes in iMACs treated with Phy or SA compared to control, presented as a heat map. (**d**) Venn diagram illustrating the overlapping peroxisomal genes both in human and mouse during OA pathogenesis.

To provide direct evidence of peroxisomal dysfunction and increasing CoA levels in osteoarthritis (OA) chondrocytes, we conducted a comprehensive analysis using public human RNA-Seq data and performed in vitro studies using iMACs. Firstly, we analyzed RNA-Seq data from the Gene Expression Omnibus (GEO) database, GSE162510 and GSE75181. In GSE162510, human chondrocytes treated with interleukin-1β (IL-1β), a cytokine known to induce inflammatory responses similar to those observed in OA, showed the significant downregulation of peroxisome-related genes. Similarly, in GSE75181, which includes RNA-Seq data from chondrocytes of OA patients, a substantial decrease in the expression of peroxisome-related genes was also observed. These findings suggest a consistent pattern of peroxisomal dysfunction in OA-affected chondrocytes. The results of pathway enrichment analysis for these datasets are presented in Figure 3a, highlighting the key metabolic and cellular processes impacted by the downregulation of peroxisome-related genes.

Next, we conducted in vitro studies using iMACs with exposure to Phy or SA, which are known to induce stress during peroxisomal metabolism, and observed that iMACs treated with Phy or SA for 24 h began to exhibit degenerative morphological changes displaying a fibroblastic morphology (Figure 3b). Consistent with the RNA-Seq data, we observed a marked decrease in the expression of most peroxisome-related genes in iMACs treated with Phy and SA (Figure 3c). This further supports that exposure to Phy or SA in iMACs induces OA pathogenesis through peroxisomal dysfunction. In addition to the downregulation of peroxisome-related genes, specific genes involved in peroxisomal acyl-CoA metabolism, such as DECR2 (2,4-dienoyl-CoA reductase 2), ECH1 (enoyl-CoA hydratase 1), EHHADH (enoyl-CoA hydratase and 3-hydroxyacyl CoA dehydrogenase), and PECR (peroxisomal trans-2-enoyl-CoA reductase), were significantly downregulated in both IL1β-treated human chondrocytes and OA patient chondrocytes. This is illustrated in Figure 3d, where the consistent downregulation of these key genes was observed across different experimental conditions and biological systems, including both human and mouse models. These results underscore the critical role of peroxisomal acyl-CoA metabolism in maintaining chondrocyte function and how its impairment may contribute to OA pathology.

The implications of these findings are profound, as they indicate that peroxisomal dysfunction and the disturbances in acyl-CoA metabolism could be pivotal in the development and progression of OA. This understanding opens up new avenues for therapeutic intervention targeting peroxisomal pathways to restore normal metabolic functions in chondrocytes. Furthermore, our study explored the potential of using MnO_2_@D-PD-coated electrodes as sensors for detecting OA conditions. Given the metabolic alterations observed, we hypothesized that these sensors could effectively monitor peroxisomal activity and CoA levels in chondrocytes. Preliminary tests demonstrated that MnO_2_@D-PD-coated electrodes were responsive to the metabolic changes induced by peroxisomal dysfunction, indicating their potential utility in diagnosing and monitoring OA. The ability to detect early metabolic changes in chondrocytes could significantly enhance the management of OA, allowing for timely intervention and improved patient outcomes.

**Figure 4:**
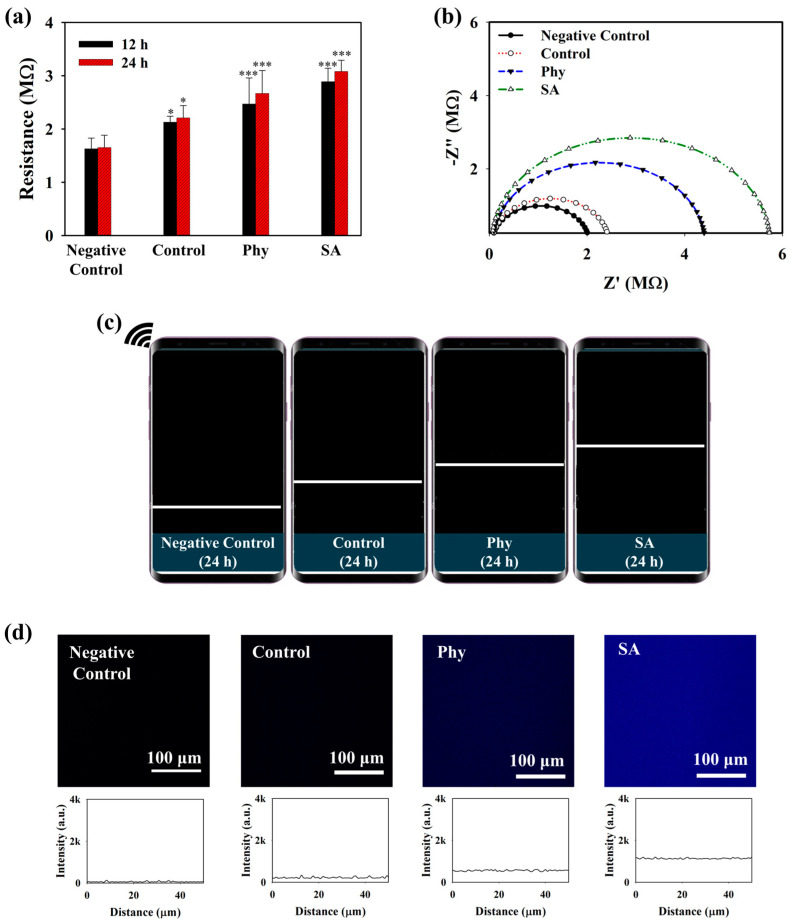
(**a**) Sourcemeter measurement (* = *p* < 0.1, *** = *p* < 0.001), (**b**) EIS spectra (24 h incubation), (**c**) wireless sensing (24 h incubation) of MnO_2_@D-PD-coated electrode, and (**d**) confocal imaging of MnO_2_@D-PD-coated electrode PET surface before (negative control) and after incubation with iMACs in control, Phy, and SA media.

The detection performance of the MnO_2_@D-PD-coated electrode for OA monitoring was evaluated using iMACs challenged with phytol (phy) or sodium acetate (SA) which are known to induce the accumulation of CoA-SH and acetyl-CoA [43,44,45,46,47]. Prior to the in vitro experiments, the acetyl-CoA levels in iMACs were measured before and after incubation with the MnO_2_@D-PD-coated electrode. As shown in Figure S4, iMACs in the control medium (Control) contained higher acetyl-CoA (2.73 nmol/mg protein) than cells incubated with the MnO_2_@D-PD-coated electrode (1.52 nmol/mg protein). This result was understandably due to the MnO_2_@D-PD on the electrode surface scavenging CoA-SH in chondrocytes; as CoA-SH plays a vital role in acetyl-CoA production in cells, the scavenging of CoA-SH would result in lower levels of acetyl-CoA [48,49]. Electrochemical responses were then compared between MnO_2_@D-PD-coated electrodes incubated with iMACs in control, Phy-challenged, and SA-challenged media. As shown in Figure 4a, the resistances of the MnO_2_@D-PD-coated electrode after incubation with iMACs in control medium were increased (R_12h_ = 2.13 MΩ, R_24h_ = 2.17 MΩ) for both 12 and 24 h incubations compared to the MnO_2_@D-PD-coated electrode without any cells (negative control). In Phy and SA media, the resistance significantly increased compared to that in the control medium and negative control, with higher-to-lower resistance trend found with SA, Phy, and control media. For the Phy medium, resistances of the MnO_2_@D-PD-coated electrodes after 12 and 24 h incubations were 2.47 and 2.67 MΩ, respectively. For the SA medium, resistances of the MnO_2_@D-PD-coated electrodes were 2.89 and 3.08 MΩ for 12 and 24 h incubations, respectively. The enhanced resistance in media from iMACs treated with Phy and SA induced an increase in the CoA-SH concentration due to the accumulation of peroxisomal CoA-SH, which further promoted more MnO_2_ cleavage [43,44]. The change in electroconductivity was also confirmed via an EIS measurement. An increasing trend in the impedance plot was clearly observed in all media groups (Control, Phy, SA) compared to the negative control, with MnO_2_@D-PD-coated electrodes incubated in Phy and SA media demonstrating more enhanced resistance compared to that for the electrode in control medium for 12 h and 24 h incubations (Figure 4b and Figure S5). Additionally, the wireless sensing system displayed a pattern of increasing signals (resistance graph), which correlated with the sourcemeter and EIS measurements (Figure 4c and Figure S6).

**Figure 5:**
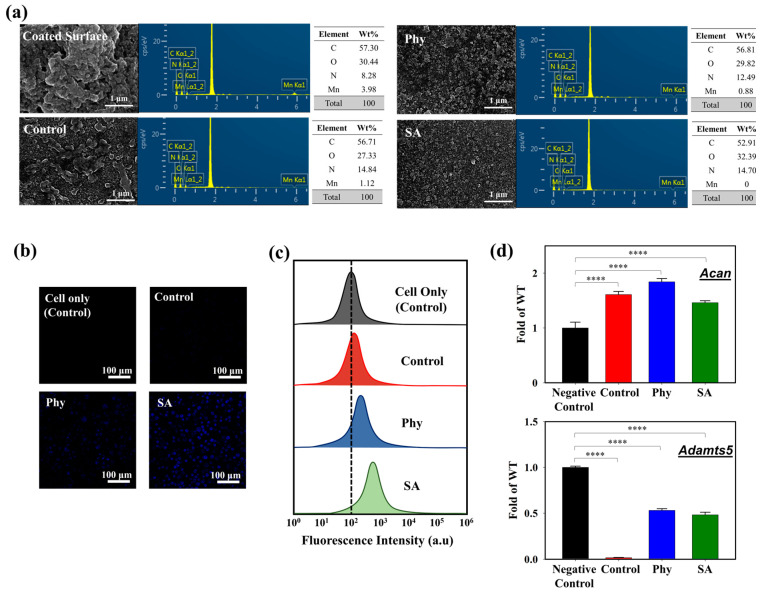
(**a**) SEM-EDX images of MnO_2_@D-PD-coated electrode surface after incubation with iMACs in control, Phy, and SA media. Cellular uptake of cells seeded on MnO_2_@D-PD-coated electrode in control, Phy, and SA media observed via (**b**) confocal microscopy and (**c**) flow cytometry. (**d**) Transcriptional level of aggrecan (*Acan*) and *Adamts5* genes in iMACs cultured in control, Phy, and SA media (**** = *p* < 0.0001).

To further evaluate the interaction of the iMACs with the MnO_2_@D-PD-coated electrodes, the fluorescence recovery of the coated surface, which would reflect the cleavage of MnO_2_, was examined after incubation in control, Phy, and SA media. The MnO_2_@D-PDs were coated onto a PET surface and incubated with cells in different media for further observations under a confocal microscope. As illustrated in Figure 4d, the intensity of the blue emission remarkably increased for surfaces treated with iMACs in control, Phy, and SA media fluorescence compared to that with control medium and the negative control, with the highest recovery of fluorescence observed in the SA medium. These results were because of MnO_2_ cleavage by CoA-SH in the cells, which reduced the FRET phenomenon on D-PD, along with a change in the surface morphology. SEM-EDX analysis clearly indicated that the surface morphology changed after the electrode was incubated with chondrocytes (Figure 5a). SEM images showed that the surface roughness of the MnO_2_@D-PD-coated electrodes changed dramatically owing to MnO_2_ cleavage after incubation with iMACs in control, Phy, and SA media. Because of the decomposition of MnO_2_, EDX elemental analysis showed that the percentage of Mn on the surface decreased after incubation with iMACs in different media, confirming the interaction between CoA-SH in the cells and MnO_2_ in the coated electrodes.

### 3.3. Cell Studies and Transcriptional Analysis of Catabolic and Anabolic Factors

The cytotoxicity of the MnO_2_@D-PD-coated electrode was evaluated via live/dead staining using confocal microscopy and flow cytometry. Based on the confocal images (Figure S7), the MnO_2_@D-PD-coated electrode showed excellent biocompatibility towards seeded iMACs after a 24 h incubation in the control, Phy, and SA media, as indicated by the prominent green emission from Annexin-V-FITC (indicating live cells) compared to the red emission from PI (indicating dead cells). Flow cytometry analysis further confirmed the low cytotoxicity of the MnO_2_@D-PD-coated electrodes, as almost 100% of the cells were alive in Phy and SA media (Figure S8). The cellular uptake of MnO_2_@D-PD by cells was also examined after incubation in each medium. Confocal images demonstrated different fluorescence intensities of MnO_2_@D-PD uptake by iMACs in each medium; cells in the SA medium exhibited the brightest blue fluorescence compared to those in Phy and control media (Figure 5b). Distinct uptake characteristics were also clearly observed via flow cytometry analysis. The data revealed that the fluorescence intensity was slightly shifted in the control group compared to that in the Phy and SA groups, with SA exhibiting the highest fluorescence intensity after MnO_2_@D-PD uptake (Figure 5c). These distinct phenomena correlate with the recovery of D-PD fluorescence after the cleavage of MnO_2_.

The transcriptional levels of catabolic and anabolic factors in articular chondrocytes were determined to understand the effect of the MnO_2_@D-PD-coated electrode on cells owing to the scavenging of CoA-SH. Aggrecan (Acan), a cartilage matrix gene in chondrocytes, is an anabolic factor suppressed in OA cartilage. Meanwhile, a disintegrin and metalloproteinase with thrombospondin motifs (Adamts)-5, a matrix-degrading enzyme, is a catabolic factor that promotes chondrocyte cell death and degrades the cartilage matrix in OA [43,44]. By analyzing the expression levels of *Acan* and *Adamts5* in articular chondrocytes after incubation with the MnO_2_@D-PD-coated electrode, the interaction between MnO_2_@D-PD and chondrocytes during detection could be determined. As shown in Figure 5d, the expression level of *Acan* in the iMACs treated with Phy and SA after incubation with the MnO_2_@D-PD-coated electrode in control media was higher than that before incubation (negative control), whereas the expression level of *Adamts5* in the iMACs after incubation was suppressed in all media compared to that before incubation. Increased levels of *Acan* and suppressed levels of *Adamts5* correlated with a decrease in acetyl-CoA or CoA-SH concentrations after interaction with the MnO_2_@D-PD-coated electrode. When CoA-SH reacts with MnO_2_ on the electrode, the CoA-SH concentration in the cells decreases, resulting in a decrease in the accumulation of acetyl-CoA. This lower accumulation of acetyl-CoA in cells further triggers the induction of *Acan* and suppression of *Adamts5*, suggesting the potential of the MnO_2_@D-PD-coated electrode for the sensitive detection of OA.

## 4. Conclusions

We successfully designed a CoA-SH-responsive dual electrochemical and fluorescence sensor based on an MnO_2_@D-PD-coated electrode that can be used for OA diagnosis. The presence of MnO_2_@D-PD on the electrode promoted sensitive redox interactions with CoA-SH in chondrocytes, producing changes in the electroconductivity and fluorescence properties of the sensor owing to the cleavage of the MnO_2_ nanosheet. The sensitivity of the MnO_2_@D-PD-coated electrode was indicated by significant changes in the resistance to various concentrations of CoA-SH, which was also verified using iMACs induced using OA conditions with the dysregulation of peroxisomal β-oxidation. The MnO_2_@D-PD-coated electrode also clearly showed distinct resistance changes when detecting chondrocyte cells in the presence of acetyl-CoA inducers in the media, such as Phy and SA (iMACs R_24 h_ = 2.67 MΩ and 3.08 MΩ, respectively), with resistance enhancement observed compared to that in the control medium (iMACs R_24 h_ = 2.17 MΩ). Moreover, owing to the reduced FRET effect after MnO_2_ cleavage, the fluorescence emission of the MnO_2_@D-PD-coated electrode significantly recovered after the addition of Phy and SA. The interaction of CoA-SH in chondrocytes with the MnO_2_@D-PD-coated electrode further altered the transcription levels of anabolic (*Acan*) and catabolic factors (*Adamts5*), which are crucial indicators of OA. Additionally, the electrochemical detection results can be monitored via a smartphone by connecting the sensor to a wireless sensing device, providing a simple system for practical applications. Based on those findings, the designed MnO_2_@D-PD-coated electrode provides excellent performance for detecting CoA-SH in OA iMACs, indicated by significant changes in the electrochemical and fluorescence properties of the electrode. This sensor also demonstrated its potential to sensitively detect OA progression based on the elevation of the CoA-SH level in the OA model induced by the acetyl-CoA inducer, as this CoA-SH-based dual signaling detection has not been explored previously. Thus, this system has the potential to be applied as a rapid and sensitive clinical diagnostic tool to support immediate decision-making for patient care and the treatment of OA in the future.

## Data Availability

The data presented in this study are available upon request from the corresponding author for privacy reasons.

## Supplementary Materials

The following supporting information can be downloaded at: https://www.mdpi.com/article/10.3390/bios14070357/s1, Figure S1: surface coatability (contact angle); Figure S2: coating stability; Figure S3: selectivity test; Figure S4: acetyl-CoA assay; Figure S5: EIS spectra (12 h incubation) of MnO_2_@D-PD-coated electrode treated with iMACs; Figure S6: wireless sensing (12 h incubation) of MnO_2_@D-PD-coated electrode treated with iMACs; Figure S7: confocal live and dead staining; Figure S8: flow cytometry analysis.
