# Supplementary material for: Electrochemical and Fluorescence MnO2-Polymer Dot Electrode Sensor for Osteoarthritis-Based Peroxisomal β-Oxidation Knockout Model
Source: Biosensors (Basel). 2024 Jul 22;14(7):357. doi: 10.3390/bios14070357 (PMC11275033; doi:10.3390/bios14070357)
Supplement: Supplementary file 1 [file biosensors-14-00357-s001.zip › biosensors-3053879-supplementary.pdf]

Supporting Information

Electrochemical and Fluorescence MnO_2-Polymer Dot Electrode Sensor for Osteoarthritis-based Peroxisomal β-Oxidation Knockout Model

Akhmad Irhas Robby[1,2†], Songling Jiang[3†], Eun-Jung Jin[3,4], Sung Young Park[1,2*]*

[1]Chemical Industry Institute, Korea National University of Transportation, Chungju 27469, Republic of Korea

[2]Department of Chemical & Biological Engineering, Korea National University of Transportation, Chungju 27469, Republic of Korea.

[3]Integrated Omics Institute, Wonkwang University, Iksan, Jeonbuk 54538, Republic of Korea

[4]Department of Biological Sciences, College of Health Sciences, Wonkwang University, Iksan, Jeonbuk 54538, Republic of Korea

***Corresponding authors:** Tel: +82-(0)43-841-5225, Fax: +82-(0)43-841-5220, E-mail: jineunjung@wku.ac.kr (Eun-Jung Jin), parkchem@ut.ac.kr (Sung Young Park)

[†]These authors are equally contributed.

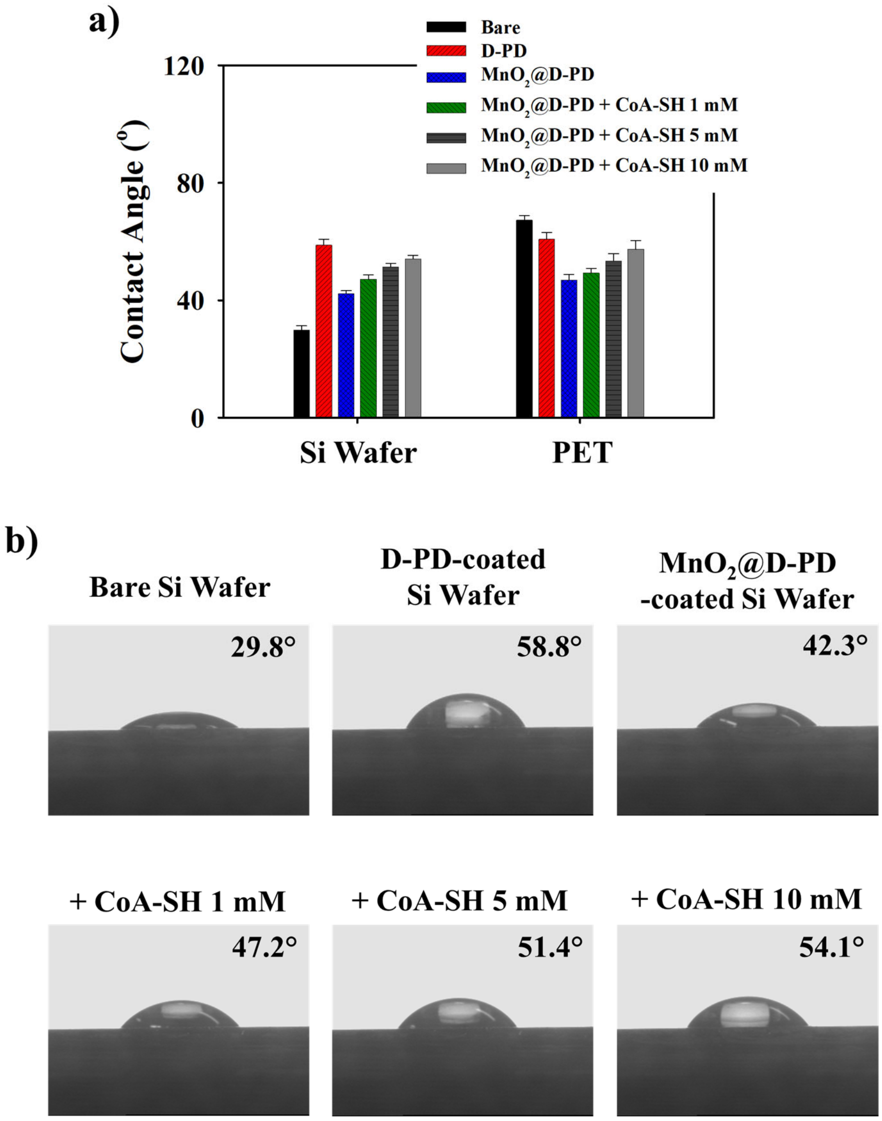

Figure S1. a) Contact angle of MnO_2@D-PD-coated surface before and after treatment with various concentrations of CoA-SH. **b)** Drop-shape pictures of water droplet angle on the MnO_2@D-PD-coated electrode (Si wafer).

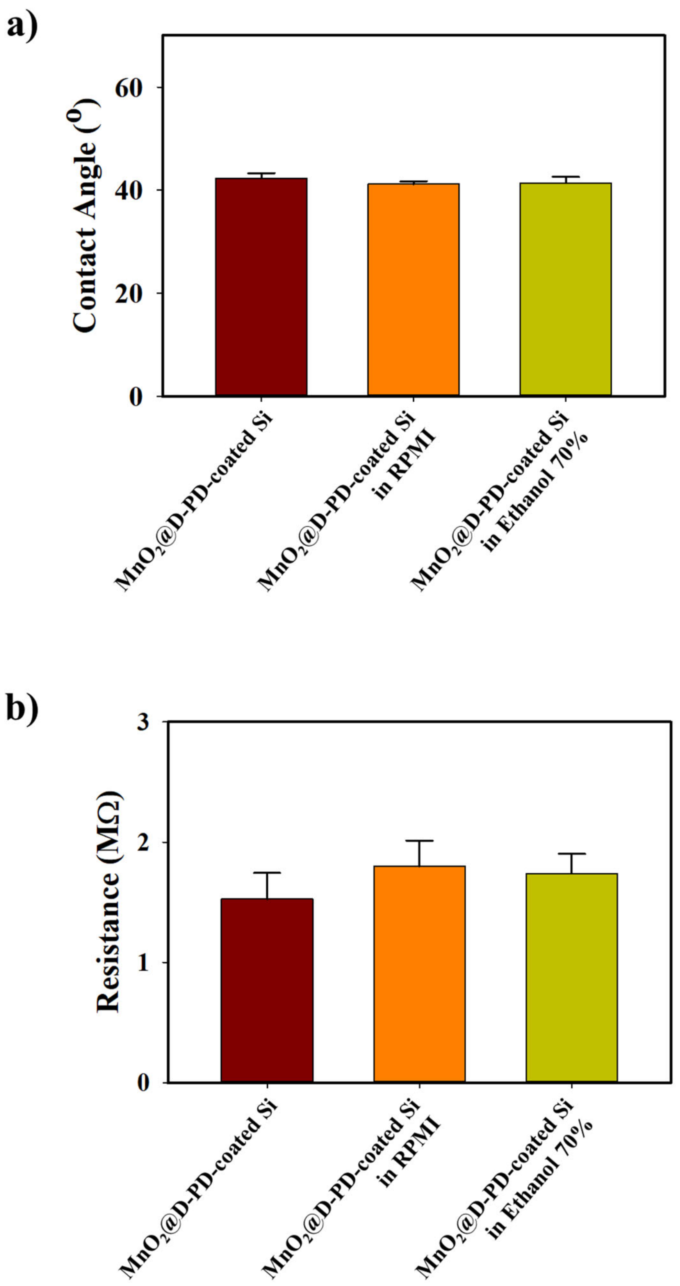

Figure S2. Stability of MnO_2@D-PD-coated surface in media (RPMI) and ethanol observed from **a)** contact angle and **b)** resistance change.

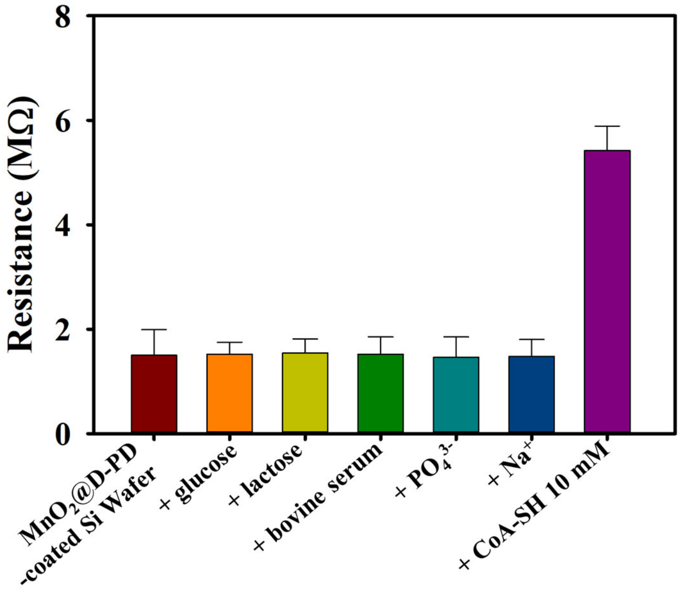

Figure S3. Selectivity of MnO_2@D-PD-coated electrode towards potential interferences.

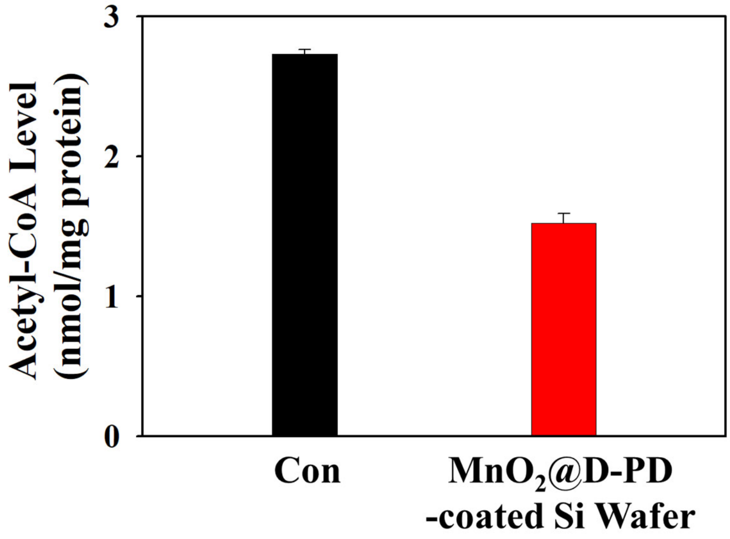

Figure S4. Acetyl-CoA assay of immature murine articular chondrocytes (iMACs) in control media (Con) and after treatment with MnO_2@D-PD-coated Si wafer.

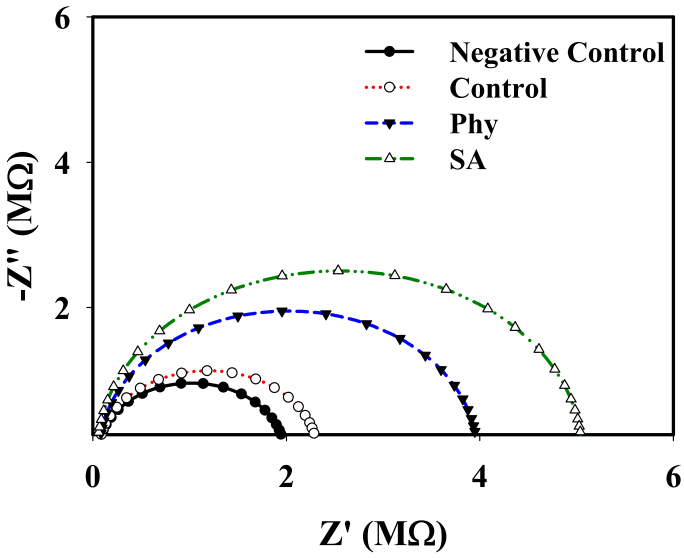

Figure S5. EIS spectra (12-h incubation) of MnO_2@D-PD-coated electrode before (negative control) and after treated with iMACs in control media (control), phytol-enriched media (Phy) and sodium acetate-enriched media (SA).

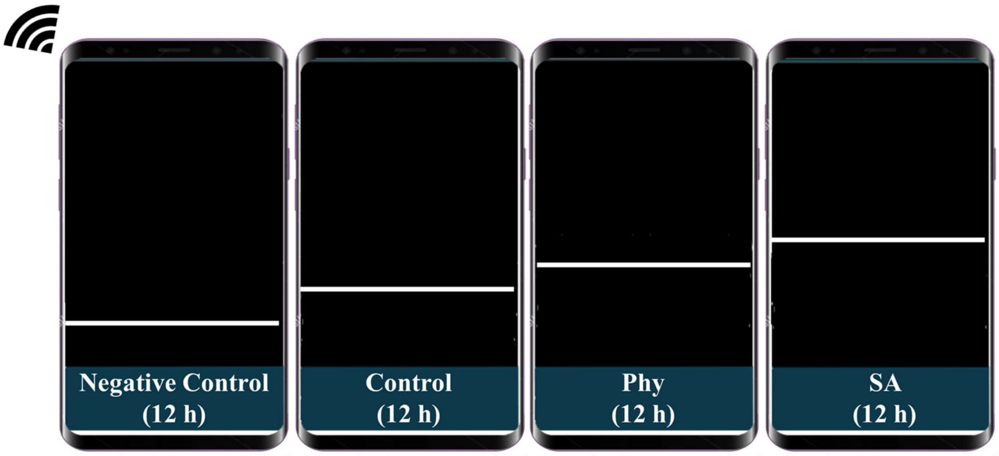

Figure S6. Wireless sensing (12-h incubation) of MnO_2@D-PD-coated electrode before (negative control) and after treated with iMACs in control media (control), phytol-enriched media (Phy) and sodium acetate-enriched media (SA).

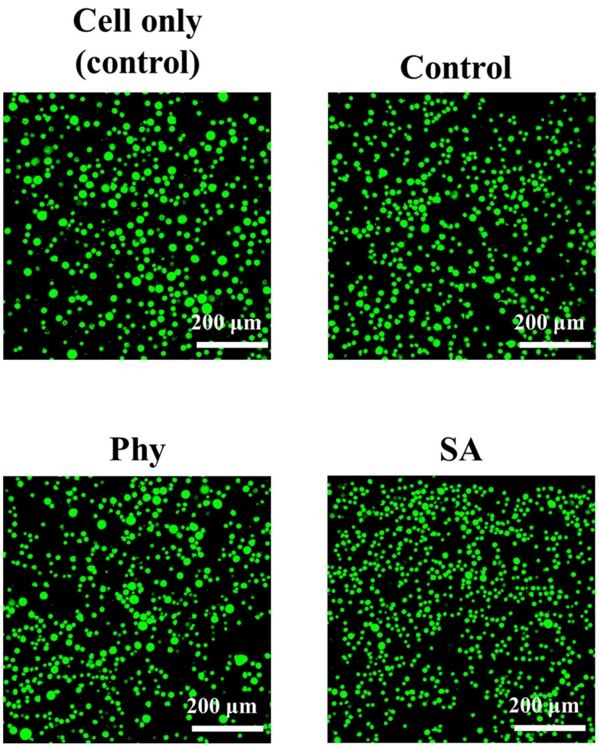

Figure S7. Live and dead assay of iMACs seeded on MnO_2@D-PD-coated electrode in control, Phy and SA-enriched media observed under confocal microscope (live staining: Annexin V-FITC, dead staining: propidium iodine/PI).

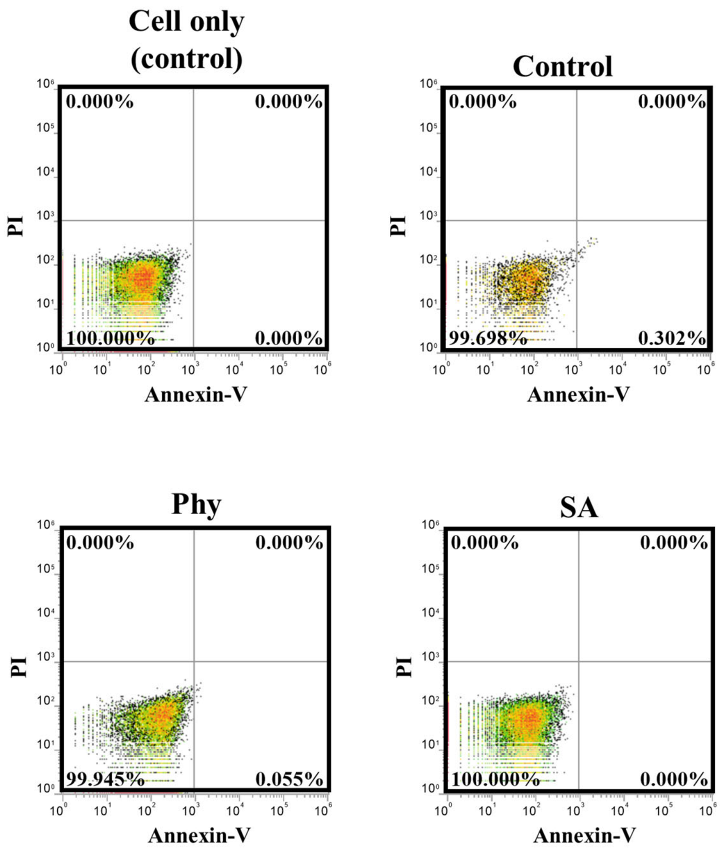

Figure S8. Live and dead assay of cell seeded on MnO_2@D-PD-coated electrode in control, Phy and SA-enriched media observed under flow cytometer (live staining: Annexin V-FITC, dead staining: propidium iodine/PI).
